# Gut microbiota in liver diseases: initiation, development and therapy

**DOI:** 10.3389/fmed.2025.1615839

**Published:** 2025-06-04

**Authors:** Jian-Xiu Yu, Jun Wu, Xin Chen, Su-gang Zang, Xue-bin Li, Li-Pei Wu, Shi-hai Xuan

**Affiliations:** Medical Laboratory Department, Affiliated Dongtai Hospital of Nantong University, Dongtai, China

**Keywords:** gut microbiota, viral hepatitis, metabolic dysfunction-associated steatotic liver disease, alcoholic fatty liver disease, liver cirrhosis

## Abstract

The gut microbiota plays a pivotal role in the pathogenesis and progression of various liver diseases, including viral hepatitis, alcoholic fatty liver disease, metabolic dysfunction-associated steatotic liver disease, drug-induced hepatitis, liver cirrhosis, hepatocellular carcinoma, and other hepatic disorders. Research indicates that dysbiosis of the gut microbiota can disrupt the integrity of the intestinal barrier and interfere with the immune functions of the gut-liver axis, thereby mediating the progression of liver diseases. Analysis of microbial composition and metabolites in fecal samples can assess the diversity of gut microbiota and the abundance of specific microbial populations, providing auxiliary diagnostic information for liver diseases. Furthermore, interventions such as fecal microbiota transplantation, probiotics, prebiotics, bacteriophages, and necessary antibiotic treatments offer multiple approaches to modulate the gut microbiota, presenting promising new strategies for the prevention and treatment of liver diseases. This review summarizes the latest research advances on the role of gut microbiota in liver diseases, offering novel theoretical foundations and practical directions for the diagnosis and treatment of hepatic disorders.

## 1 Introduction

Liver diseases have emerged as a significant global health challenge, encompassing various conditions such as viral hepatitis, metabolic dysfunction-associated steatotic liver disease (MASLD), alcoholic liver disease (ALD), and hepatocellular carcinoma (HCC) (1). According to GLOBOCAN 2023 statistics, over 800 million people worldwide are affected by liver diseases, with approximately 2 million deaths annually, accounting for 4% of global mortality (2, 3). The gut microbiota, the largest microbial community in the human body, comprises more than 50 phyla and approximately 1,500 different species, playing a crucial role in maintaining health and contributing to disease pathogenesis (4). In healthy individuals, the gut microbiota is predominantly composed of beneficial bacteria such as *Bacteroidetes* and *Firmicutes*, along with smaller proportions of *Proteobacteria*, *Actinobacteria*, and *Verrucomicrobia* (5). Recent studies have demonstrated that gut microbiota dysbiosis is closely associated with the development and progression of various liver diseases, including chronic hepatitis B (CHB), ALD, MASLD, liver cirrhosis (LC), and HCC (6). The underlying mechanisms involve gut-liver axis signaling, metabolite regulation, and immune microenvironment remodeling, among others (7–10). Despite advancements in diagnostic and therapeutic technologies for liver diseases, significant challenges remain in diagnostic methods, criteria, and therapeutic targets, highlighting the need for further improvements.

The gut microbiota and liver diseases are closely interconnected through the gut-liver axis. Nutrients, bacterial metabolites, and potential harmful substances in the gut can enter the liver via the portal venous system (11, 12). Simultaneously, the liver delivers metabolites such as bile acids (BA) to the intestinal lumen through the biliary tract, thereby regulating the composition and function of the gut microbiota (13). Additionally, liver diseases can disrupt intestinal barrier function, promote gut microbiota dysbiosis, and further impair the normal function of the gut-liver axis. Dysbiosis of the gut microbiota may exacerbate inflammatory and fibrotic processes in liver diseases, aggravating liver injury.

With advancing research, beyond traditional antiviral and immunomodulatory therapies, modulating the gut microbiota through fecal microbiota transplantation (FMT), probiotics, and specific dietary interventions has emerged as a novel adjunctive approach for liver disease treatment (14, 15). This review summarizes the role of the gut microbiota in the onset, progression, diagnosis, and pathogenesis of liver diseases, and explores the impact of therapeutic strategies aimed at restoring gut microbiota balance on disease progression, thereby providing new insights and methods for the diagnosis and treatment of liver diseases. Furthermore, this review analyzes the specific manifestations of the gut microbiota in different liver diseases, discusses its potential as a diagnostic biomarker in liver diseases, elucidates the mechanisms of microbiota in disease pathogenesis, and evaluates the potential of microbiota-targeted therapies in the management of liver diseases.

## 2 Expression characteristics, mechanistic research, and diagnostic-therapeutic applications of gut microbiota in viral hepatitis

In 2022, the World Health Organization (WHO) revised the 2030 strategy for the comprehensive elimination of viral hepatitis, initially adopted in 2016, setting forth more specific and quantifiable targets (16). Viral hepatitis, a significant global health burden, is primarily caused by five viruses: hepatitis A virus (HAV), hepatitis B virus (HBV), hepatitis C virus (HCV), hepatitis D virus (HDV), and hepatitis E virus (HEV) (17). The Global Hepatitis Report 2024 disclosed that the number of deaths from viral hepatitis increased from 1.1 million in 2019 to 1.3 million in 2022, with 83% attributable to hepatitis B and 17% to hepatitis C, resulting in approximately 3,500 deaths daily worldwide from these two types of hepatitis (18). Recent studies have indicated a correlation between gut microbiota dysbiosis and the occurrence of viral hepatitis, suggesting that hepatitis virus infections can alter the diversity of the gut microbiome (9, 10).

### 2.1 Alteration of gut microbiota in HBV

Chronic hepatitis B (CHB) patients exhibit characteristic alterations in gut microbiota: multiple 16S rRNA sequencing studies demonstrate a significant increase in *Firmicutes* abundance and a decrease in *Bacteroidetes*, resulting in an elevated Firmicutes/*Bacteroidetes* (F/B) ratio (19, 20). However, geographical variations may influence this trend, as one study observed reduced *Firmicutes* and increased *Bacteroidetes* in CHB patients (21). Further analysis reveals three distinct enterotypes in CHB patients, dominated by *Bacteroides*, *Blautia*, and *Prevotella*, respectively (8). Notably, fecal samples from occult HBV-infected individuals show a marked depletion of butyrate-producing *Faecalibacterium* coupled with an abnormal enrichment of opportunistic pathogen *Subdoligranulum* (22). LEfSe analysis confirms differential regulation of 19 genera in HBV-infected individuals, including upregulated *Alloprevotella* and downregulated *Bacteroides* (17). Most concerningly, HBV-related liver disease patients exhibit a pro-inflammatory microbiome signature characterized by opportunistic pathogens (e.g., *Proteus*, *Klebsiella*) enrichment and butyrate-producing bacteria (e.g., *Ruminococcus*) depletion (23).

### 2.2 Alteration of gut microbiota in HCV

In 86 patients with HCV infection, the abundance of 10 taxa, including *Desulfovibrio*, *Eubacterium eligens*, and *Prevotella*, was significantly higher than that in the HC group, while the abundance of 11 genera, such as *Barnesiella*, *Colidextribacter*, and *Dorea*, was significantly reduced (17). Additionally, treatment-naïve HCV patients exhibited increased gut microbiota diversity, with elevated abundances of *Prevotella*, *Megasphaera*, and *Ruminococcaceae*, and decreased abundances of *Bacteroides*, *Streptococcus*, and *Enterobacteriaceae* (24). 16S RNA sequencing analysis also revealed lower bacterial diversity in 166 Japanese patients with chronic hepatitis C (CHC), characterized by a reduction in the order *Clostridiales* and an increase in *Streptococcus* and *Lactobacillus* (25). Compared to the HC group, the total abundance of *Lactobacillus* and *Lactobacillus acidophilus* was significantly lower in patients with chronic HCV infection (26).

### 2.3 Alteration of gut microbiota in HEV, HAV, and HDV

In 33 patients with acute hepatitis E (AHE), the abundance of *Proteobacteria*, *Gammaproteobacteria*, and *Enterobacteriaceae* was significantly higher in the gut compared to the HC group (27). Furthermore, compared to the AHE group, the HEV-associated acute liver failure (ALF) group showed increased abundances of *Gammaproteobacteria*, *Proteobacteria*, *Xanthomonadaceae*, and *Stenotrophomonas*, and decreased abundances of *Firmicutes*, *Streptococcus*, *Subdoligranulum*, and *Lactobacillus* (28). HAV, an acute and self-limiting disease, has limited research on gut microbiota changes during infection. 16S rRNA analysis revealed gut microbiota dysbiosis in HIV patients co-infected with HAV, characterized by reduced *Proteobacteria* abundance and enrichment of *Bifidobacterium* and *Bacteroides*, with this dysbiosis persisting long after clinical recovery (29). As for HDV infection, no relevant studies on gut microbiota have been identified, likely because HDV is an incomplete virus requiring HBV for replication, making it challenging to obtain relevant data (17). These findings suggest that regional, dietary, and ethnic differences may contribute to the variability in gut microbiota expression in viral hepatitis-related liver diseases (Table 1). Therefore, long-term, multicenter studies are still needed to further explore the relationship between gut microbiota and viral hepatitis.

**Table 1:** Manifestations of gut microbiota in the disease process of viral hepatitis.

Studies	Subjects	Increased	Decreased
Lin et al. (19)	CHB (*n* = 58)	**Phylum level**: *Firmicutes*, *Verrucomicrobia*, *Fusobacteria* **Genus level**: *Streptococcus*, *Blautia*, *Veillonella*, *Fusobacteria*, *Akkermansia*	**Phylum level**: *Bacteroidetes* **Genus level**: *Bacteroides*, *Megamonas*, *Bacteroides*, *Sutterella*, *Lachnoclostridium*
Zeng et al. (21)	CHB (*n* = 21)	**Phylum level**: *Bacteroidetes* **Family level**: *Enterobacteriaceae* **Genus level**: *Bacteroides*, *Prevotella*, *Atopobium*, *Veillonella*, *Alistipes*	**Phylum level**: *Firmicutes* **Family level**: *Bifidobacterium*, *Clostridiaceae*
Zhang et al. (8)	CHB (*n* = 110)	**Genus level**: *Bacteroides*, *Blautia*, *Prevotella*	/
Yang et al. (17)	HBV (*n* = 546)	**Genus level**: Nineteen genera, including *Alloprevotella*, *Butyricimonas*, *Colidextribacter*	**Genus level**: *Bacteroides*, *Parabacteroides*, *Sutterella*
Zhang et al. (8)	CHB Advanced fibrosis (*n* = 52)	**Genus level**: *Escherichia coli*	**Genus level**: *Alistipes shahii*, *Alistipes obesi*, *Blautia stercoris*, *Desulfovibrio piger*, *Roseburia hominis*, *Ruminococcus callidus*
Wang et al. (34)	HBV-ACLF (*n* = 212)	**Genus level**: *Enterococcus*, *Pediococcus*, *Janthinobacterium*, *Faecalibacterium*, *Clostridiaceae*, *Phascolarctobacterium*	/
Yao et al. (35)	HBV-ACLF (*n* = 91)	**Phylum level**: *Firmicutes*, *Proteobacteria*, *Actinomycetota* **Genus level**: *Veillonella*, *Streptococcus*, *Enterococcus*, *Fusobacteria*, *Klebsiella*	**Phylum level**: *Bacteroidetes* **Genus level**: *Bacteroides*, *Ruminococcus*, *Butyricimonas*, *Lachnospiraceae*, *Sutterella*
Yang et al. (17)	HCV (*n*=86)	**Genus level**: *Desulfovibrio*, *Eubacterium eligens*, and *Prevotalla*	**Genus level**: *Alloprevotella*, *Butyricimonas*, *Barnesiella*, *Colidextribacter*, *Dorea*
Sultan et al. (24)	HCV (*n* = 38)	**Family level**: *Ruminococcaceae* **Genus level**: *Prevotella*, *Succinivibrio*, *Catenibacterium*, *Megasphaera*	**Family level**: *Enterobacteriaceae*, *Erysipelotrichaceae*, *Rikenellaceae* **Genus level**: *Bacteroides*, *Dialister*, *Bilophila*, *Streptococcus*, *Parabacteroides*, *Alistipes*
Inoue et al. (25)	CHC (*n* = 166)	**Family level**: *Enterobacteriaceae* **Genus level**: *Streptococcus*, *Lactobacillus*, *Bacteroides*	**Order level**: *Clostridiales*
Wu et al. (27)	AHE (*n* = 33)	**Phylum level**: *Proteobacteria* **Class level**: *Gammaproteobacteria* **family level**: *Enterobacteriaceae*, *Xanthomonadaceae*	**Family level**: *Bifidobacteriaceae*

### 2.4 The diagnostic value of microbiota in viral hepatitis

The gut microbiota plays a pivotal role in the progression, early detection, and diagnosis of HBV-related liver diseases, with compositional shifts serving as both prognostic indicators and diagnostic biomarkers (30). A case–control study utilizing Culturomics technology identified Enterocloster bolteae as a novel microbial signature in CHB patients, distinguishing them from healthy controls (31). In treatment-naïve CHB populations, HBeAg positivity correlates with specific taxonomic enrichments: HBeAg+ individuals exhibit elevated levels of *Eubacterium coprostanoligenes*, *Christensenellaceae*_R_7, *Oscillospirales*_UCG_010, and *Haemophilus*, paralleled by reduced *Erysipelatoclostridium* and *Lachnoclostridium* abundance compared to HBeAg- counterparts (32). Notably, these phyla-level differences remain statistically insignificant despite genus-level alterations. Antiviral therapy significantly reshapes microbial profiles. Tenofovir alafenamide-treated HBeAg+ patients demonstrate marked increases in *Bacteroidetes*, *Prevotella*, *Alistipes*, *Oxalobacter*, and *Butyricicoccaceae*_UCG_009, coupled with depletion of *Proteobacteria*, *Actinobacteria*, and *Bifidobacterium* species (29). These changes contrast with immune phase-specific biomarkers, where *Ruminococcus gnavus* and *Akkermansia muciniphila* differentiate immune-tolerant from immune-active CHB states (33). Microbial dynamics correlate with clinical outcomes across disease spectra. In HBV-ACLF, metagenomic analysis reveals *Enterococcus* enrichment correlates with disease progression, while *Faecalibacterium* dominance associates with recovery (34). *Bacteroidetes* abundance inversely correlates with serum AFP levels, whereas *Veillonella* shows positive association with total bilirubin (TBIL), and *Coprococcus* demonstrates dual correlations with TBIL/INR (negative) and prothrombin time (positive) (35). Antiviral intervention partially restores microbial balance, particularly in *Blautia*, *Dorea*, and *Ruminococcaceae*_UCG-013 populations (36). Complications such as portal hypertension and hepatic encephalopathy (HE) exhibit microbial predictors. TIPS therapy preserves microbial diversity in HBV-related portal hypertension, though HE-free patients show superior microbiota synergy compared to post-TIPS HE cases (37). In ACLF, *Blautia*, *Coprococcus*, and *Methanobrevibacter* abundance inversely correlates with coagulopathy and jaundice severity (37). Cross-viral comparisons reveal conserved and disease-specific patterns. HCV patients demonstrate post-treatment enrichment of *Coriobacteriaceae* and *Staphylococcaceae* with concurrent *Morganellaceae* reduction (38), while HEV severity correlates with *Lactobacillaceae* and *Gammaproteobacteria* abundance (28). ROC analysis identifies *Butyricimonas*, *Escherichia-Shigella*, and *Veillonella* as potential progression markers for viral hepatitis (AUC > 0.700) (17), with five HCV-specific OTUs achieving AUC > 0.710 (24) (Table 2). Despite these advances, the field lacks standardized biomarkers. Longitudinal multicenter studies remain essential to validate microbial signatures, clarify causal mechanisms, and establish microbiota-based diagnostics for viral hepatitis management.

**Table 2:** Gut microbiota-clinical correlations in liver diseases.

Studies	Disease type	Significantly altered gut microbiota	Associated clinical indicators/functions	Correlation/diagnostic value
Yao et al. (35)	HBV-ACLF	*Bacteroidetes*	AFP	Negative correlation
		*Veillonella*	TBIL	Positive correlation
		*Coprococcus*	TBIL, INR	Negative correlation
Zhao et al. (37)	HBV-ACLF	*Methanobrevibacter*, *Blautia*, *Coprococcus*	Jaundice, coagulation dysfunction	Negative correlation
Shen et al. (36)	CHB	*Turicibacter*, *Adlercreutzia*	AST	Negative correlation
		*Streptococcus*	TBIL, DBIL, HBV-DNA	Positive correlation
Ashour et al. (26)	HCV	Total lactate, *Lactobacillus acidophilus*	HCV-RNA	Negative correlation
Wu et al. (28)	HCV	*Lactobacillaceae*, *Gammaproteobacteria*	INR, Th cell	Positive correlation
Yang et al. (17)	Viral hepatitis	*Butyricimonas, Escherichia-Shigella*, *Lactobacillus and Veillonella*	Disease progression	AUC > 0.700
Ganesan et al. (64)	ALD	*Proteobacteria*, *Fusobacteria*	Disease severity	Positive correlation
		*Bacteroidota*	Disease severity	Negative correlation
Zhong et al. (71)	Alcoholic cirrhosis	*Streptococcaceae*, *Streptococcus, Veillonella*	Severity of liver injury	Positive correlation
Addolorato et al. (73)	Alcohol use disorder	*Akkermansia*	Bacterial translocation and inflammatory response	Negative correlation
Park et al. (72)	ELE/LC/HCC	Signature microbial consortium	ML	AUC = 0.940–0.970
Lee et al. (101)	MASLD	*Ruminococcaceae*, *Dorea*	Fibrosis severity	Negative correlation
Caussy et al. (104)	MASLD	27 signature microbial species	Diagnostic value	AUC = 0.920
Alboraie et al. (91)	MASLD	*Fusobacteria*, *Veillonellaceae*	Disease progression	Positive correlation
		*Rikenellaceae*, *Barnesiellaceae*, *Adolescentis*	Disease progression	Negative correlation
Yan et al. (23)	HBV-LC	*Lachnospiraceae*, *Ruminococcaceae*	Protective effect	Negative correlation
		*Enterococcaceae*, *Staphylococcaceae*	Pathogenicity	Positive correlation
Zhang et al. (45)	LC	*Akkermansia*, *Barnesiella*	Proinflammatory response	AUC = 0.824
Cao et al. (192)	LC	*Bifidobacterium*	HBV-DNA	Negative correlation
Efremova et al. (128)	LC	*Negativicutes*, *Enterobacteriaceae*, *Veillonella*, *Klebsiella*	TNF-α, IL-6	Positive correlation
Yang et al. (17)	HBV-LC	*Butyricimonas*, *Veillonella*, *Escherichia-Shigella*	Diagnostic value	AUC = 0.917, 0.797, and 0.794, respectively
Yang et al. (46)	HCC	Nine characteristic bacterial genera including *Elizabethkingia*, *Klebsiella*, and *Stenotrophomonas*	Diagnostic value	AUC = 0.810
		Combined with serum AFP levels	Diagnostic value	AUC = 0.980
Ren et al. (39), Peng et al. (150)	HCC	30 microbial biomarkers	Diagnosis of vascular invasion	AUC = 0.806

### 2.5 Mechanisms of the gut microbiota in viral hepatitis

The gut microbiota plays a crucial role in metabolic processes, not only facilitating the digestion and absorption of food but also producing various metabolites that influence host metabolic functions. During HBV infection, bacteria from the *Leptospiraceae* family may exert a positive role in managing HBV infection by reducing bacterial translocation and lowering lipopolysaccharide (LPS) levels (39). Multi-omics analysis has demonstrated that electroacupuncture combined with tenofovir disoproxil fumarate can increase the abundance of gut microbiota such as *Bacteroides* and *Blautia* by modulating the PPAR signaling pathway, while enhancing the expression of tight junction proteins (ZO-1, Occludin, Claudin-4), thereby improving intestinal barrier integrity (40). Additionally, *Enterocloster bolteae* isolated from chronic HBV patients can produce ethanol, potentially promoting the progression of liver disease (31). *Ruminococcus gnavus* promotes cholic acid production by secreting bile salt hydrolase, which activates the farnesoid X receptor alpha (FXRα) signaling pathway. This process enhances the transcription of HBV core antigen (HBcAg), thereby prolonging the HBV immune tolerance phase. Conversely, *Akkermansia muciniphila* suppresses *Ruminococcus gnavus* growth and its bile acid-converting function through metabolite secretion, reduces CA levels, blocks the FXRα-HBcAg axis, and facilitates HBV clearance (33). The reduction of BA in viral hepatitis is associated with increased intestinal permeability, leading to elevated levels of LPS and other endotoxins, which promote the progression of liver disease (41). In an LPS-treated mouse model of HBV replication, gut microbiota dysbiosis triggers endotoxemia, inducing Kupffer cells to produce IL-10 and enhancing Kupffer cell-mediated T cell suppression, which plays a critical role in HBV persistence (42). Depletion of gut microbiota impairs systemic anti-HBV humoral and cellular immune responses, resulting in delayed clearance of HBV antigens (43). Furthermore, bacterial extracts derived from HBV-CLD patients stimulate peripheral blood mononuclear cells, potentially promoting fibrotic progression by altering peripheral immune responses (elevated Th17 and reduced Th1), which adversely affects patient prognosis (36).

HCV infection drives disease progression by inducing alterations in the intestinal bile acid profile and gut microbiota dysbiosis, which downregulate CYP8B1 expression (a key enzyme in cholic acid biosynthesis), thereby perpetuating pathogenesis through the gut-microbiome-liver axis (44). Increased circulating LPS levels in CHC patients indicate that microbial translocation is closely linked to hepatic inflammation and injury, thereby driving disease progression (45). Additionally, impaired intestinal barrier function in HCV patients is evidenced by elevated levels of zonulin-1, LPS, and calprotectin, suggesting that intestinal inflammation, microbial imbalance, and increased barrier permeability play significant roles in the pathophysiology of HCV infection (46). These studies demonstrate that the pathogenesis of viral hepatitis is closely related to intestinal barrier function, microbiota-derived metabolites, and BA metabolism. Viral infections can alter the diversity and composition of gut microbiota, leading to gut-liver axis dysregulation and exacerbating hepatic inflammation and injury. Therefore, modulating gut microbiota may emerge as a novel strategy to improve intestinal barrier function and mitigate liver disease progression. Metabolites and microbiota signatures may serve as potential biomarkers for disease diagnosis, though their clinical application requires further validation. Future research should focus on elucidating the specific mechanisms of gut microbiota in liver diseases to enhance clinical diagnosis and treatment efficacy.

### 2.6 Treatment of the gut microbiota in viral hepatitis

#### 2.6.1 FMT

Currently, targeting the gut microbiota has emerged as a novel therapeutic approach for viral hepatitis infections and their complications. FMT as a method to restore and reconstruct the balance and diversity of gut microecology, has demonstrated promising outcomes. In a study involving 20 patients with liver disease related to CHB progression, FMT treatment significantly improved the Shannon and Simpson indices of gut microbiota, repaired the impaired abundance of gut microbiota, and subsequently promoted the improvement of amino acid metabolism (47). In a preliminary study in China, FMT induced HBeAg clearance in 18 HBeAg-positive patients who had undergone long-term antiviral therapy (48). Similarly, in a non-randomized pilot clinical trial involving 14 CHB patients in India, the potential safety and efficacy of FMT in achieving viral suppression and HBeAg clearance in HBeAg-positive CHB patients were observed (49). Furthermore, a study by Suez et al. found that the benefits of probiotics might be counteracted by the restoration of the intestinal mucosa following antibiotic use, whereas autologous FMT could rapidly and nearly completely restore the intestinal mucosa within days after administration (50). This suggests that autologous FMT or the development of personalized probiotic approaches may help achieve intestinal mucosal protection without interfering with the antibiotic-induced disruption of host microbiome recolonization (50).

#### 2.6.2 Direct-acting antiviral

In studies targeting HCV, the goal of antiviral therapy is to eradicate HCV, mitigate associated liver damage, and ultimately achieve a cure (51). A 72-week DAA treatment study (*n* = 50) demonstrated significant recovery of microbial diversity, particularly enriching short-chain fatty acid (SCFA)-producing genera (*Blautia*, *Bifidobacterium*, *Subdoligranulum*, and *Fusicatenibacter*) while reducing microbial translocation markers like lipopolysaccharide-binding protein (LBP) (52). Other studies have also demonstrated that DAA treatment increases microbial diversity, alters bacterial abundance, and benefits intestinal health in patients with HCV-related chronic liver disease (53, 54). However, existing research findings exhibit some inconsistencies. A 12-week trial (*n* = 42) found quantitative but not qualitative microbiota alterations, providing important insights into the complex relationship between CHC and gut microbiota dysbiosis (38). In contrast, a study by Ponziani et al. found that 12 HCV-related cirrhosis patients exhibited significant changes in their overall gut microbial composition after 1 year of DAA treatment, characterized by a reduction in the abundance of potentially pathogenic bacteria such as *Enterobacteriaceae*, *Enterococcus*, and *Staphylococcus* (55). These discrepancies may be attributed to factors such as sample size and the timing of fecal sample collection. Additionally, studies on the impact of DAA treatment on gut microbiota diversity in CHC patients have yielded divergent results. A prospective study in Germany observed an increase in gut microbiota diversity among non-cirrhotic patients following treatment (56), whereas a prospective study in Taiwan found no significant differences between cirrhotic and non-cirrhotic patient subgroups (57). These inconsistencies may be attributed to confounding factors such as ethnicity and dietary habits, highlighting the need for larger-scale and more rigorously designed studies to further elucidate the impact and mechanisms of DAA treatment on gut microbiota.

#### 2.6.3 Probiotics, farnesoid X receptor ligands, and bacteriophages

Several studies have also explored the potential roles of probiotics, FXR ligands, and bacteriophages in treating HCV infection and its complications. HCV-infected patients administered heat-treated *Enterococcus faecalis* strain FK-23 exhibited a significant reduction in serum AST levels without adverse side effects (58). CHC patients taking probiotics containing *Lactobacillus acidophilus* and *Bifidobacterium* showed a 25% improvement in response to interferon IFN-α and ribavirin therapy (59). DCA, an FXR ligand, may overcome BA metabolic disturbances and ameliorate CHC progression when supplemented directly or through probiotics that convert cholic acid to DCA (44). Additionally, correcting gut microbiota dysbiosis in HCV-infected individuals using specific bacteriophages targeting relevant bacteria has been proposed (60) (Table 3). Although these studies suggest that maintaining intestinal barrier integrity, correcting gut microbiota dysbiosis, preventing microbial translocation, and further reducing chronic inflammation may serve as novel strategies for treating HCV infection and its complications, current research remains limited by small sample sizes and a lack of randomized controlled trial designs. Therefore, the efficacy and safety of these approaches require further validation through large-scale clinical trials. Future research should focus on elucidating the underlying mechanisms and optimizing individualized treatment regimens to enhance clinical utility.

**Table 3:** Applications and mechanisms of microbial-based interventions in liver diseases.

Studies	Subjects	Microbial interventions	Mechanisms of action	Evidence level
Inoue et al. (44), Zhang et al. (45), Yang et al. (46)	CHB	FMT	Restores microbial diversity, modulates amino acid metabolism, and inhibits viral replication	Clinical study
Chauhan et al. (49), Suez et al. (50), Chinese Society of Hepatology and Chinese Society of Infectious Diseases; Chinese Medical Association (51)	HCV	DAA	Enhances microbial diversity and increases beneficial bacteria abundance	Clinical study
Neag et al. (41), Ponziani et al. (55)	HCV	Probiotics	Reduces AST levels and ameliorates bile acid metabolic disorders	Clinical study, animal model
Brown et al. (76)	ALD	Diet	Increases *Bacteroides acidifaciens* abundance and alleviates liver injury	Animal model
Shen et al. (79), Yi et al. (81), Liu et al. (82)	ALD	Probiotics	Modulates gut microbiota, repairs intestinal barrier, and reduces alcohol-induced oxidative stress, lipid accumulation, and hepatic inflammation	Animal model
Wang et al. (80)	ALD	Probiotics	Mitigates hepatic injury	Clinical study
Vatsalya et al. (83)	ALD	FMT	Improves short-/mid-term survival rates and clinical severity scores	Clinical study
Niu et al. (84)	ALD	FMT	Reverses liver injury by regulating arachidonic acid and retinol metabolism pathways	Animal model
Ma et al. (109), Zhen et al. (110)	MASLD	Diet	Reduces advanced fibrosis risk in MASLD and prevents MASH progression	Clinical study
Mao et al. (111), Matsumoto et al. (112)	MASLD	Probiotics	Ameliorates metabolic syndrome and prevents hepatic steatosis/injury	Animal model
Gallage et al. (113)	MASLD	Probiotics	Reduces body weight and intrahepatic fat content	Clinical study
Li et al. (115)	MASLD	Synbiotics	Decreases steatosis/fibrosis severity with parallel reduction in hepatic injury markers and inflammatory mediators	Clinical study
Zafar et al. (132), Maslennikov et al. (133)	LC	Probiotics	Increases beneficial/harmful bacteria ratio, with significant improvement in liver function and inflammatory cytokines	Clinical study
Huang et al. (130), Kang et al. (137)	LC	FMT	Improves duodenal mucosal diversity, corrects dysbiosis, and enhances cognitive function in cirrhotic patients	Clinical study
Bloom et al. (140)	LC	FMT	Enhances gut microbiota α-diversity (richness/evenness) and reduces hepatic inflammation in cirrhotic rats	Animal model
Hong et al. (141)	LC	Engineered carbon	Alleviates liver injury, fibrosis progression, and mortality in ACLF models	Animal model
Bloom et al. (140), Wang et al. (156), Tilg et al. (157)	HCC	FMT	Restores microbial diversity and gut barrier integrity, markedly reducing hepatic inflammation and fibrosis	Animal model
Hu et al. (159)	HCC	Probiotics	Modulates microbiota, stabilizes intestinal barrier, and reduces carcinogenic toxicity	Animal model
Zhen et al. (110)	HCC	Diet	Improves MASH and fibrosis severity	Animal model

## 3 Expression characteristics, mechanistic research, and diagnostic-therapeutic applications of gut microbiota in ALD

### 3.1 Alteration of gut microbiota in ALD

ALD, driven by chronic excessive alcohol intake, progresses from hepatic steatosis to fibrosis and cirrhosis via gut microbiota dysbiosis (61–63). Clinical and animal studies consistently show ALD-associated microbial shifts: increased *Proteobacteria* and decreased *Bacteroidota* in patients (64), while murine models reveal ethanol exposure duration-dependent changes—short-term (9 h) ethanol gavage elevates *Bacteroidota*/*Parabacteroides* (65), whereas prolonged intake (10–14 days) increases *Firmicutes* and *Akkermansia* (66, 67). Alcohol reduces anti-inflammatory taxa (*Muribaculaceae*, *Bacteroides*) (68), promotes pathogenic overgrowth (*Enterococcus*, *Alistipes*) (69), and disrupts intestinal permeability. Despite elevated *Enterococcus faecalis* in alcoholic hepatitis patients (*n* = 75), its abundance lacks correlation with disease severity or mortality (70) (Table 4). These findings highlight the need for larger human cohorts to reconcile interspecies discrepancies and validate therapeutic targets.

**Table 4:** Manifestations of gut microbiota in the disease process of ALD.

Studies	Subjects	Increased	Decreased
Ganesan et al. (64)	ALD patients (*n* = 185)	**Phylum level**: *Proteobacteria* **Genus level**: *Fusobacterium*, *Lactobacillus*, *Bifidobacterium*, *Hemophilus*, *Staphylococcus*, *Streptococcus*, *Akkermansia*	**Phylum level**: *Bacteroides* **Genus level**: *Prevotella*, *Alistipes*, *Bacteroides*, *Parabacteroides*, *Phascolarctobacterium*, *Faecalibacterium*
Smirnova et al. (193)	ALD patients (*n* = 34)	**Genus level**: **Phylum level**: *Firmicutes*, *Proteobacteria* **Family level**: *Enterobacteriaceae*, *Lachnospiraceae*, *Lactobacillaceae*, *Prevotellaceae*, *Saccharibacteria*, *Streptococcaceae*, *Veillonellaceae*	**Phylum level**: *Bacteroidetes* **Family level**: *Acidaminococcaceae*, *Bacteroidaceae*, *Erysipelotrichaceae*, *Lachnospiraceae*y, *Peptococcaceae*, *Peptostreptococcaceae*, *Porphyromonadaceae*, *Prevotellaceae*, *Rikenellaceae*, *uminococcaceae*, *Sutterellaceae*
Lang et al. (194)	ALD patients (*n* = 74)	**Genus level**: *Veillonella*, *Enterococcus*	**Genus level**: *Akkermansia*
Wang et al. (65)	ALD rats (*n* = 12)	**Phylum level**: *Bacteroidota*, *Proteobacteria* **Genus level**: *Muribaculum*, *Enterococcus*, *Bacteroides*, *Parasutterella*	**Phylum level**: *Firmicutes* **Genus level**: *Lactobacillus*, *Enterobacter*, *Bacteroides*, *Faecalibaculum*
Grander et al. (195)	ALD rats (*n* = 20)	**Genus level**: *Muribaculaceae*, *Fecalibactulum*	**Genus level**: *Enterobacteriaceae*, *Roseburia*, *Clostridium*
Zhang et al. (196)	ALD rats (*n* = 10)	**/**	**Genus level**: *Akkermansia*, *Bacteroides*
Wang et al. (197)	ALD rats (*n* = 10)	**Phylum level**: *Proteobacteria*, *Patescibacteria* **Genus level**: *Bacteroides*, *Enterobacter*, *Escherichia-Shigella*	**Phylum level**: *Firmicutes*, *Epsilonbacteraeota*, *Actinobacteria*, *Tenericutes*, *Cyanobacteria* **Family level**: *Lachnospiraceae NK4A136* **Genus level**: *Desulfovibrio*
Yin et al. (198)	ALD rats (*n* = 5)	**Genus level**: *Helicobacter* sp, *Pichia kudriavzevii*	**Genus level**: *Faecalibaculum rodentium*
Li et al. (199)	ALD rats (*n* = 9)	**Family level**: *Bacteroidaceae*, *Erysipelotrichaceae*, Sutterellaceae **Genus level**: *Bacteroides*, *Parabacteroides*, *Parasutterella*	**Family level**: *Muribaculaceae*, *Lachnospiraceae* **Genus level**: *Lactobacillus*

### 3.2 The diagnostic value of microbiota in ALD

Long-term alcohol consumption significantly alters the diversity and composition of gut microbiota, which greatly contributes to the progression of ALD. Studies have shown that the overall structure of gut microbial communities varies significantly across different stages of ALD. Advanced ALD is marked by *Proteobacteria*/*Fusobacteria* enrichment and *Bacteroidota* depletion, with *Streptococcus* dominance emerging as a potential biomarker for liver injury severity (71). Metagenomic analysis further confirms that the abundance of *Proteobacteria* increases while that of *Bacteroidota* decreases with the progression of ALD severity (64). Additionally, analysis of gut microbiota data from 263 ALD patients including elevated liver enzymes (ELE), cirrhosis, and HCC using a machine learning (ML) strategy revealed that the ML strategy achieved diagnostic AUC values of 0.940, 0.970, and 0.960 for ELE, LC, and HCC, respectively, indicating the significant diagnostic value of gut microbiota in ALD (72). In patients with alcohol use disorder, a notable feature of gut microbiota is the reduction of *Akkermansia* and the increase of *Bacteroides*. These changes are closely associated with bacterial translocation, inflammatory responses, and enhanced functions of the γ-aminobutyric acid metabolic pathway and energy metabolism, potentially further driving the progression of alcohol-related liver disease (73). Current research highlights the critical role of gut microbiota in the onset and progression of ALD, although the specific mechanisms remain to be further explored. Future studies should integrate multi-omics technologies (e.g., metagenomics, metabolomics) and advanced methods such as machine learning to delve deeper into the relationship between gut microbiota and ALD, and to validate its potential as a diagnostic biomarker.

### 3.3 Mechanisms of the gut microbiota in ALD

The pathogenesis of ALD has not been fully elucidated and is currently believed to involve multiple factors, including alcohol and its metabolites, gut microbiota dysbiosis, oxidative stress, and gut-liver axis dysfunction (74). Alcohol disrupts the gut-liver axis at multiple levels, including altering the composition of the gut microbiome, impairing mucus and epithelial barrier functions, and suppressing the production of antimicrobial peptides. These changes increase the translocation of microbes and their metabolites, thereby exacerbating the pro-inflammatory environment in the liver (75). Excessive alcohol consumption disrupts the balance of gut microbiota and affects the metabolism of intestinal contents, such as SCFAs, indoles, and BAs, which play critical roles in various physiological and pathological processes and directly influence the progression of ALD (76). Gut bacteria exacerbate hepatic inflammatory injury in ALD by disrupting the intestinal barrier, activating pattern recognition receptors in the liver, altering the metabolism of luminal contents such as BAs, indoleacetic acid, and SCFAs, and producing bacterial exotoxins (e.g., cytolysin) (74). Alcohol induces oxidative damage in hepatocytes by promoting reactive oxygen species generation and inhibiting antioxidant enzyme activity, while blocking Nrf2 nuclear translocation and the expression of its downstream antioxidant genes, thereby weakening hepatic antioxidant defenses; gut microbiota dysbiosis exacerbates hepatic oxidative stress, creating a vicious cycle that highlights the critical role of Nrf2-Keap1 signaling pathway inhibition in ALD progression (77, 78). Current research indicates that the pathogenesis of ALD involves complex interactions among multiple factors and pathways. Future studies should further focus on the interplay between gut microbiota and host metabolism, particularly the specific mechanisms of metabolites such as SCFAs, BAs, and indoles in ALD. Integrating multi-omics technologies and animal model studies will help comprehensively elucidate the pathogenesis of ALD and provide a theoretical basis for developing combined strategies based on gut microbiota modulation and antioxidant therapy.

### 3.4 Treatment of the gut microbiota in ALD

#### 3.4.1 Diet

In recent years, interventions targeting gut microbiota, including dietary modifications, probiotic supplementation, FMT, and bacteriophage therapy, have demonstrated significant efficacy in improving ALD in numerous animal experiments and clinical trials. A diet rich in soluble dietary fiber increases the abundance of *Bacteroides acidifaciens*, thereby alleviating alcohol-induced liver injury in mice (79). Additionally, *Solanum nigrum* L. berry extract significantly ameliorates alcoholic liver injury by modulating gut microbiota, lipid metabolism, inflammation, and oxidative stress (80). Trilobatin, a novel natural food additive, exhibits potential for preventing and treating ALD by regulating the microbiota-gut-liver axis and the YAP/Nrf2 pathway (81).

#### 3.4.2 Probiotics

Probiotics have garnered considerable attention for their role in improving ALD. *Lactobacillus rhamnosus* NKU FL1-8 reduces alcohol-induced oxidative stress, lipid accumulation, and hepatic inflammation by modulating gut microbiota and repairing the intestinal barrier (82). In clinical studies, a 6-month treatment with *Lactobacillus rhamnosus* GG significantly reduced liver injury and alcohol consumption in patients with moderate to severe alcoholic hepatitis (83). Furthermore, both viable and inactivated *Lactobacillus paracasei* CCFM1120 V and D effectively protect the liver from ethanol-induced damage by altering gut microbiota composition, strengthening the intestinal barrier, and enhancing hepatic antioxidant capacity (84). Lactic acid bacteria (LAB) strains have also been shown to directly alleviate ALD symptoms, including reducing inflammatory cytokines, inhibiting fatty liver, and restoring gut microbiota dysbiosis (85).

#### 3.4.3 FMT

FMT, as an emerging therapy, has demonstrated significant efficacy in ALD treatment. Studies indicate that FMT improves short- and medium-term survival rates and clinical severity scores in patients with severe alcoholic hepatitis (86). In animal models, FMT reverses alcohol-induced liver injury by ameliorating gut microbiota dysbiosis and modulating metabolic pathways such as arachidonic acid and retinol metabolism (87). Bacteriophage therapy specifically targeting *Enterococcus faecalis* has shown promising results in mouse models, reducing cytolysin in the liver and significantly improving ALD (70) (Table 3). However, this therapy still requires validation in larger-scale prospective clinical trials to confirm its relevance in humans.

In a mouse model of acute alcohol exposure, *Musculus senhousei* peptide intervention exerts protective effects by mitigating gut-liver axis injury and reversing abnormalities in oxidative stress and inflammation-related biomarkers (88). Additionally, lentinan demonstrates significant hepatoprotective effects by reducing hepatic steatosis, alleviating oxidative stress and inflammatory responses, and promoting the proliferation of antioxidant probiotics (89). These findings suggest that interventions targeting gut microbiota hold broad application prospects in ALD treatment.

However, most current studies are limited to animal models or small-scale clinical trials. Future large-scale, multicenter prospective studies are needed to validate the efficacy and safety of these approaches.

## 4 Expression characteristics, mechanistic research, and diagnostic-therapeutic applications of gut microbiota in MASLD

### 4.1 Alteration of gut microbiota in MASLD

MASLD, formerly known as non-alcoholic fatty liver disease, is a chronic liver condition affecting approximately 30% of the global population (90). Characterized by abnormal lipid accumulation in hepatocytes, MASLD can progress from simple steatosis to metabolic dysfunction-associated steatohepatitis (MASH), and ultimately lead to hepatic fibrosis, cirrhosis, and even hepatocellular carcinoma (91). Studies have shown that the diversity and abundance of gut microbiota are significantly reduced in MASLD patients compared to HC (92). However, findings vary across regions and ethnicities. For example, a study in Indonesia involving 37 MASLD patients reported a predominance of *Firmicutes* and an elevated F/B ratio (93). In contrast, a study in Taiwan involving 50 biopsy-confirmed MASLD patients demonstrated a higher abundance of *Bacteroidetes*, a lower abundance of*Firmicutes*, a reduced F/B ratio, and decreased levels of *Ruminococcaceae*, *Clostridiales*, and *Clostridium* compared to healthy individuals (94). A study in Korea involving 23 MASLD patients with elevated liver enzymes found an enrichment of *Firmicutes*, an increased F/B ratio, and a significant rise in the abundance of *Veillonella*, *Dialister*, *Collinsella*, *Latilactobacillus*, and *Bifidobacterium* (95). These inconsistencies in F/B ratios may be attributed to differences in dietary habits across countries. Furthermore, animal model studies support the role of gut microbiota in MASLD. In mouse models of MASLD induced by a high-fat diet (HFD) or Western diet, the F/B ratio was significantly elevated (96). In MASLD patients, the abundance of beneficial bacteria such as *Akkermansia muciniphila*, *Faecalibacterium prausnitzii*, and *Bifidobacterium* is significantly reduced (97). A study involving 100 adolescent MASLD patients found a notable decrease in the abundance of *Lactobacillus* and *Escherichia coli* and a significant increase in *Prevotella* (98). Metagenomic sequencing analysis further revealed an increased abundance of *Bacteroidetes* and a reduced abundance of 11 genera, including *Alistipes*, *Barnesiella*, and *Eisenbergiella*, in MASLD patients (99). These findings indicate that the gut microbiota of MASLD patients exhibits significant diversity, which may be closely related to factors such as ethnicity, dietary habits, and geographic environment.

### 4.2 The diagnostic value of microbiota in MASLD

Numerous studies have elucidated the critical role of the gut microbiome in the progression of MASLD (100). The gut microbiome critically influences MASLD progression through compositional and functional alterations. Non-obese MASLD patients exhibit reduced *Ruminococcaceae* abundance compared to obese counterparts, with its depletion correlating with fibrosis severity (101), while protective *Dorea* shows similar depletion patterns (102). Pathogenic *Klebsiella pneumoniae* (identified in 60% of Chinese MASLD patients) directly drives MASLD development via alcohol production, as validated by FMT experiments (103). Distinct microbial signatures differentiate disease stages: *Streptococcus* enrichment occurs in both MASLD and MASLD-cirrhosis groups, but *Megasphaera* is exclusive to cirrhosis (104). Diagnostic models integrating 27 bacterial features achieve high accuracy (AUC = 0.920) for cirrhosis detection (104). Metabolic shifts are evident in MASLD, characterized by ethanol-producing bacteria (*Enterobacteriaceae*, *Megasphaera*) dominance and SCFA-producing taxa (*Ruminococcus*, *Eggerthellaceae*) reduction (105). Advanced fibrosis associates with *Methanobrevibacter* depletion and *Slackia* enrichment (106). Machine learning identifies 12 MASLD-linked taxa, including *Fusobacteria* (positive correlation) and *Rikenellaceae* (negative correlation) (91) (Table 2). These findings underscore gut microbiota’s role in MASLD severity stratification and prognostic prediction.

### 4.3 Mechanisms of the gut microbiota in MASLD

**Figure 1:**
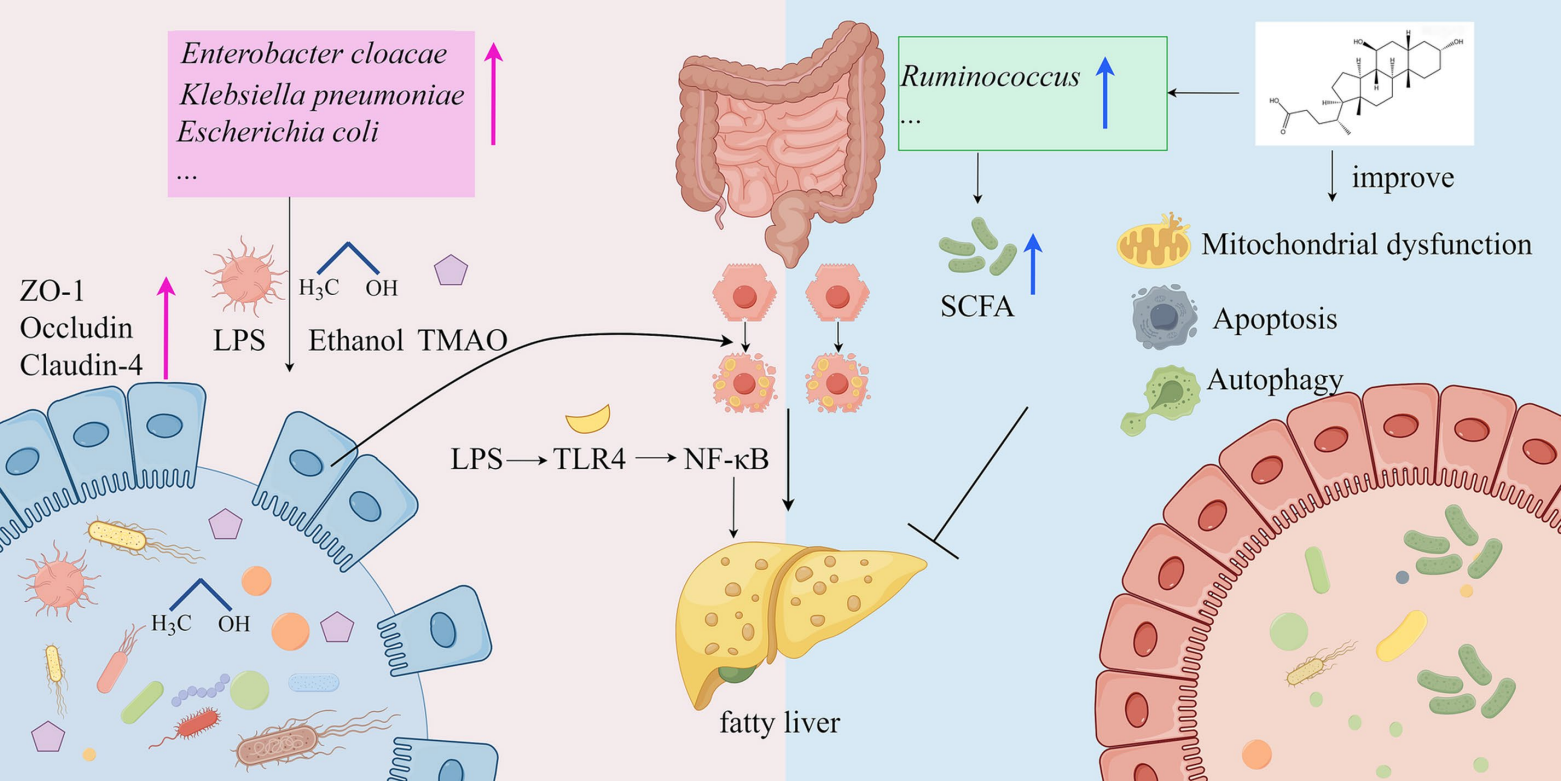
Mechanisms of the gut microbiota in the development of MASLD (by Figdraw). LPS, lipopolysaccharide; ZO-1, Zonula occludens-1; TMAO, Trimethylamine-N-oxide; TLR4: Toll-like Receptor 4; NF-KB, Nuclear Factor kappa-light-chain-enhancer of activated B cells; SCFA, short-chain fatty acid.

The onset of MASLD is closely associated with dysfunction of the gut microbiome, primarily involving two mechanisms: abnormal elevation of intestinal barrier permeability and imbalance of microbiota-derived metabolites (107). Trimethylamine-N-oxide (TMAO), a microbiota-derived metabolite, disrupts tight junction proteins to impair intestinal barrier integrity while directly promoting hepatic lipid accumulation in both cellular (HepG2) and rodent models (108). This process is driven by gut bacterial conversion of trimethylamine (TMA) to TMAO via hepatic FMO3, with clinical studies confirming serum TMAO levels as a biomarker of steatosis severity (109, 110). Concurrently, pathogenic bacteria such as *Klebsiella pneumoniae* exacerbate MASLD through alcohol-mediated steatosis and inflammation (103), while Gram-negative overgrowth (e.g., *Enterobacter cloacae* B29) triggers TLR4/NF-κB signaling via endotoxin release (107). Contrastingly, protective microbiota like *Ruminococcus* attenuate lipid deposition through SCFA production and 7α-dehydroxylase regulation (102). Bile acid metabolism is further modulated by gut microbes, with ursodeoxycholic acid (UDCA) enhancing autophagy and mitochondrial function to restore host-microbiota equilibrium (111). These findings suggest that the role of gut microbiota in MASLD is dualistic, encompassing both disease-promoting factors and potential protective mechanisms (Figure 1).

### 4.4 Treatment of the gut microbiota in MASLD

#### 4.4.1 Diet

Currently, a balanced diet and healthy lifestyle are considered the best strategies for improving MASLD. The high intake of soy and its products, fish, shellfish, and seaweed in the Japanese diet is significantly associated with a reduced risk of advanced fibrosis in MASLD patients (112), likely due to the anti-inflammatory and antioxidant components abundant in these foods. Additionally, intermittent fasting (e.g., the 5:2 regimen) can effectively prevent the development of MASH and improve diagnosed MASH and fibrosis without affecting total caloric intake (113), suggesting that dietary pattern modulation may have unique intervention effects on MASLD.

#### 4.4.2 Probiotic

In animal models, MASLD mice induced by HFD showed significant reductions in body weight, triglycerides, total cholesterol, and low-density lipoprotein cholesterol levels after supplementation with *Lactiplantibacillus plantarum* DSR33 (114). Furthermore, the abundance of *Bacteroides thetaiotaomicron* (B. theta) is positively correlated with the alleviation of metabolic syndrome in both early and late stages of MASLD. HFD-fed mice supplemented with B. theta for 12 weeks exhibited reduced body weight and fat accumulation, improved hyperlipidemia and insulin resistance, and prevention of hepatic steatohepatitis and liver injury (115). These findings suggest that specific bacterial strains may exert therapeutic effects by modulating host metabolism and inflammatory responses. A randomized double-blind study showed that 68 obese MASLD patients experienced significant reductions in body weight and intrahepatic fat after 12 weeks of supplementation with a probiotic mixture (including *Lactobacillus acidophilus*, *Lactobacillus rhamnosus*, *Lactobacillus paracasei*, *Pediococcus pentosaceus*, *Bifidobacterium lactis*, and *Bacillus breve*) (116). However, in another PROBILIVER clinical trial, 44 biopsy-confirmed MASH patients showed no significant improvement in serum liver enzymes, transient elastography, MASLD fibrosis score, or fatty liver index after 24 weeks of probiotic mixture supplementation (117). This discrepancy may be related to the selection of probiotic strains, dosage, treatment duration, and individual patient differences.

#### 4.4.3 Synbiotics and Postbiotics

Additionally, synbiotics (a combination of probiotics and prebiotics) have shown promise in MASLD treatment. A randomized double-blind clinical trial demonstrated that 50 MASLD patients had significant reductions in steatosis and fibrosis, along with decreased levels of liver injury markers and inflammatory mediators, after 28 weeks of synbiotic supplementation (118). Notably, the probiotic *Bifidobacterium adolescentis* alleviates steatohepatitis by inhibiting lipid peroxidation and NF-κB activation and mitigates non-alcoholic fatty liver through the production of SCFAs (119). Moreover, postbiotics (bacterially derived functional compounds) exhibit significant hepatoprotective properties by enhancing intestinal barrier function, modulating gut microbiota composition, optimizing lipid metabolism, and reducing liver inflammation and steatosis (120) (Table 3).

These studies indicate that dietary adjustments, supplementation with probiotics, prebiotics, or synbiotics positively reshape gut microbiota composition and enhance its activity, thereby improving liver function damage in MASLD patients. However, current research findings remain inconsistent, and future large-scale, long-term clinical trials are needed to validate the efficacy of different intervention strategies and further explore their mechanisms of action.

## 5 Expression characteristics, mechanistic research, and diagnostic-therapeutic applications of gut microbiota in LC

### 5.1 Alteration of gut microbiota in LC

LC represents a severe stage of chronic liver disease characterized by extensive hepatocyte degeneration, fibrosis, and nodular regeneration, leading to significant morbidity and mortality (121, 122). In hepatitis B-related cirrhosis, a common subtype of LC, progressive liver damage is further aggravated by gut microbiota dysbiosis and associated metabolic dysfunction (123). Studies consistently demonstrate that LC patients exhibit markedly reduced gut microbial diversity compared to healthy individuals (39, 124), with notable depletion of beneficial bacteria such as *Agathobacter* and *Prevotella_9* alongside overgrowth of opportunistic pathogens including *Streptococcus* (124, 125). This dysbiotic pattern becomes particularly pronounced in HBV-related cirrhosis patients progressing toward hepatocellular carcinoma, where protective bacterial families like *Lachnospiraceae* and *Ruminococcaceae* diminish while potentially harmful *Enterobacteriaceae* and *Staphylococcaceae* proliferate (23). Geographic variations in microbiota profiles have been observed, with Chinese LC patients showing elevated *Bacteroidota/Firmicutes* ratios and *Proteobacteria* abundance correlated with inflammatory responses (45), while North American cohorts demonstrate distinct associations between *Enterobacteriaceae/Streptococcaceae* dominance and clinical outcomes including extrahepatic organ failure (126). Emerging diagnostic approaches utilizing microbial signatures, such as machine learning models based on 14 differential bacterial genera, show promising accuracy for LC detection (AUC 0.824) (45). The clinical relevance of these microbial alterations is underscored by their correlations with disease complications, including the association between *Akkermansia muciniphila* depletion and sarcopenia development (127), as well as the close relationship between pathogenic bacterial overgrowth and systemic inflammatory markers like TNF-α and IL-6 (128) (Table 2). These findings collectively establish gut microbiota dysbiosis as a key contributor to LC progression through multiple interconnected pathways.

### 5.2 Mechanisms of the gut microbiota in LC

**Figure 2:**
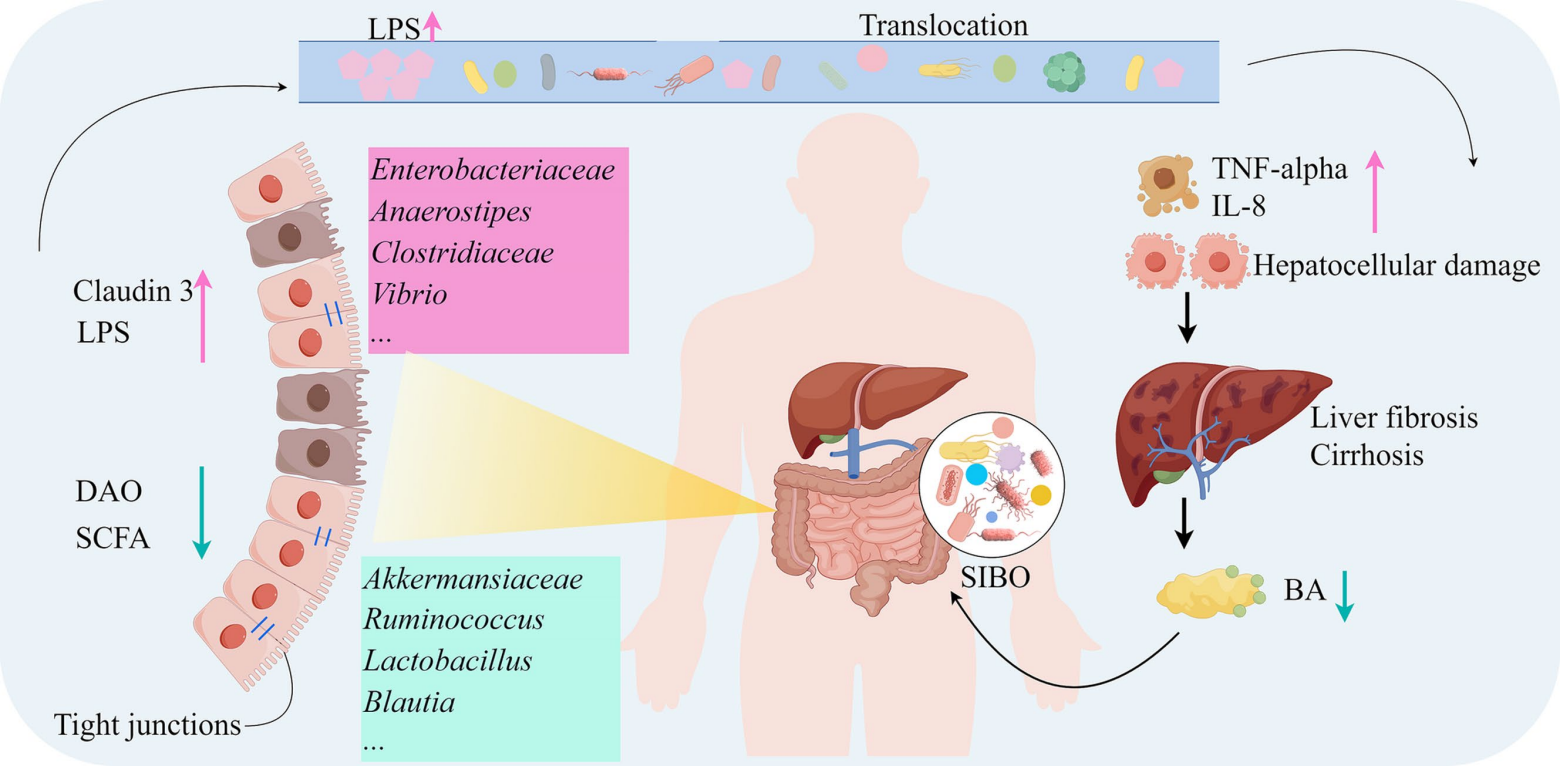
Mechanisms of the gut microbiota in the development of LC (by Figdraw). DAO, Diamine oxidase; BA, Bile acids; IL-8, Interleukin-8; SIBO, Small intestinal bacterial overgrowth; TNF-alpha, Tumor necrosis factor-alpha.

The gut microbiota plays a critical role in the pathogenesis of LC. In LC patients, gut microbiota dysbiosis, bacterial overgrowth, and increased intestinal permeability disrupt the protective mechanisms of the gut, leading to pathological bacterial translocation and increased endotoxin uptake. These endotoxins subsequently reach the liver and mesenteric lymph nodes, activating immune cells and triggering the release of pro-inflammatory cytokines such as TNF-α and IL-8 (129). Meta-analysis results indicate that endotoxin-producing *Enterobacteriaceae* and *Enterococcus* are significantly increased in LC patients, which may be related to the impaired intestinal mucosal barrier function caused by LC (130). Gut microbiota dysbiosis leads to intestinal barrier injury (DAO reduction, Claudin-3 dysfunction), bacterial toxin translocation (LPS elevation) triggers systemic inflammation (TNF-α), forming a vicious gut-liver axis cycle that exacerbates liver fibrosis and portal hypertension (125). In LC patients, reduced liver function and decreased BA secretion result in bacterial overgrowth and changes in microbiota composition, weakening the inhibition of potential pathogenic microorganisms and exacerbating intestinal inflammation and mucosal barrier damage (131). Small intestinal bacterial overgrowth (SIBO) can also trigger bacterial translocation and endotoxemia, activating chronic liver inflammation and promoting liver fibrosis (132). Moreover, SIBO is closely associated with hyperdynamic circulation and other hemodynamic changes in cirrhosis patients, potentially serving as a primary factor in inducing these changes through systemic inflammation (133). The impaired intestinal barrier and gut microbiota dysbiosis not only lead to bacterial translocation and endotoxemia but also exacerbate liver injury and fibrosis through abnormal related metabolites. In LC patients, dysbiosis of the ascending colon mucosa-associated microbiota, particularly the reduction of SCFA-producing bacteria, compromises intestinal barrier integrity and BA metabolism, thereby exacerbating liver fibrosis progression via the gut-liver axis. This microbial imbalance correlates with downregulated FGF19 expression and upregulated profibrogenic factors (e.g., TGF-β1), establishing a vicious cycle (134). Additionally, the overgrowth of *Viridans streptococci* may induce hyperammonemia in CHC and LC patients (25). The gut microbiota plays a pivotal role in the development of cirrhosis, with its dysbiosis not only directly affecting liver inflammation and fibrosis but also influencing systemic metabolism and immune responses through the gut-liver axis (Figure 2).

### 5.3 Treatment of the gut microbiota in LC

#### 5.3.1 Probiotics

Targeting gut microbiota dysbiosis may serve as an effective strategy for preventing and slowing the progression of liver cirrhosis. In a study involving 160 patients with HBC, the administration of the ready-to-eat supplement *Lactobacillus paracasei* N1115 significantly increased gut microbiota diversity, elevated the proportion of beneficial bacteria such as *Bacteroides* and *Bifidobacterium*, and reduced the proportion of harmful bacteria such as *Escherichia*, *Shigella*, and *Streptococcus*. Concurrently, liver function indicators improved significantly, and levels of inflammatory factors decreased markedly (135). A meta-analysis further supported these findings, with 22 included randomized controlled trials demonstrating that probiotics can enhance intestinal permeability and liver toxin filtration capacity, significantly reducing gamma-glutamyl transferase, AST, blood ammonia, and endotoxin levels (136). Additionally, animal experiments and human tissue analyses confirmed that the probiotic *Akkermansia muciniphila* can restore levels of pro-inflammatory cytokines, endotoxins (LPS and LBP), serotonin-related cognitive function, and liver injury, making it a potential therapeutic candidate for alleviating liver fibrosis and cognitive impairment symptoms (137). Four single-arm trials (58 participants) and two randomized controlled trials (66 participants) showed that the use of probiotics significantly reduced hepatic venous pressure gradient (138). Another randomized controlled trial indicated that a multifactorial intervention consisting of multi-strain probiotics, home-based exercise, and branched-chain amino acids improved frailty in cirrhosis patients and reduced emergency visits and fall incidents (139).

#### 5.3.2 FMT

Furthermore, after oral administration of FMT capsules, the abundance of SCFA-producing bacteria (*Bifidobacterium adolescentis* and *Bifidobacterium angulatum*) increased in LC patients, positively correlating with improvements in HE psychological scores, suggesting that FMT has a beneficial effect on cognitive function in LC patients (140). However, in decompensated cirrhosis patients, defects in immune status may reduce the therapeutic efficacy of FMT (141). Despite this, oral FMT capsules enriched with *Lachnospiraceae* and *Ruminococcaceae* demonstrated good safety and tolerability in LC patients and improved duodenal mucosal diversity and dysbiosis (142). Animal experiments also confirmed that FMT increased the abundance of beneficial bacteria such as *Lactobacillaceae* and *Bacteroidaceae*, significantly improving gut microbiota diversity, richness, and evenness in cirrhotic rats, reducing liver inflammation, and thereby ameliorating liver fibrosis and cirrhosis (143).

#### 5.3.3 Engineered carbon

In a LC mouse model, the engineered carbon bead adsorbent Yaq-001 positively influenced microbial composition and metabolism by reducing intestinal permeability, significantly alleviating liver injury, fibrosis progression, and mortality in ACLF animals, and achieved primary endpoints for safety and tolerability in clinical trials, providing strong preclinical theoretical and safety support for LC patients (144) (Table 3).

Probiotics, FMT, and engineered carbon beads improve gut microbiota dysbiosis through different mechanisms, reduce inflammation, enhance liver function, and positively impact cognitive function, offering new approaches for the treatment of cirrhosis patients. However, these methods each have unique advantages and limitations. Future research could further explore the combined application and optimized integration of these methods to enhance treatment efficacy and patient quality of life.

## 6 Expression characteristics, mechanistic research, and diagnostic-therapeutic applications of gut microbiota in HCC

### 6.1 Alteration of gut microbiota in HCC

HCC is the fourth leading cause of cancer-related deaths globally, with its incidence primarily associated with hepatitis B (40%), hepatitis C (40%), alcohol (11%), and MASH (2). According to GLOBOCAN data, there were 905,677 new cases and 830,180 deaths from liver cancer worldwide in 2020 (145). Recent studies have shown that alterations in the gut microbiota are closely related to the occurrence and progression of HCC (146). In early-stage HCC patients, the species richness of fecal microbiota is increased compared to the LC group (39, 124). Analysis of published fecal datasets from four different regions in China revealed that the relative abundance of *Firmicutes* was significantly lower in HCC patients compared to HC, and further decreased with disease progression, while the relative abundance of *Bacteroidetes* and *Proteobacteria* significantly increased (45). However, Yan et al. reported inconsistent findings in a study conducted in Beijing, showing that the abundance of both *Bacteroidetes* and *Firmicutes* gradually decreased in HCC patients (23). Additionally, the abundance of *Proteobacteria*, *Streptococcus*, and *Ruminococcus* was significantly higher in the HCC group compared to controls, while the abundance of *Subdoligranulum* was significantly reduced (46). In early-stage HCC patients, the abundance of *Actinobacteria* increased, and 13 genera, including *Gemmiger* and *Parabacteroides*, were enriched in early HCC (39). The relative abundance of potentially beneficial bacteria, such as *Lactobacillus*, *Bifidobacterium*, and *Bacteroides*, was significantly reduced in HCC patients, while the relative abundance of potentially pathogenic bacteria, such as *Escherichia-Shigella* and *Enterococcus*, was significantly increased (147). Furthermore, *Akkermansia* was most enriched in LC patients, while its abundance was relatively lower in the HC group, CHB patients, and HCC patients (21). In conclusion, to gain a more comprehensive understanding of the specific role and expression characteristics of gut microbiota in HCC, multicenter, large-sample clinical trials are still needed for further exploration.

### 6.2 The occurrence and early warning value of metabolites in HCC

Gut microbiota-derived metabolites play a pivotal role in HCC progression and early detection. Comparative analyses reveal significant enrichment of *Proteobacteria* and *Actinobacteria* in advanced liver disease (HCC and cirrhosis) versus HC, with *Escherichia-Shigella*, *Veillonella*, and *Streptococcus* consistently elevated at the genus level (15). Viral-related HCC patients exhibit increased *Faecalibacterium* and *Coprococcus*, while non-hepatitis C-related cases show *Bacteroides* and *Ruminococcus* dominance (148). Early postoperative recurrence is associated with higher *Dialister*, *Veillonella*, and *Bifidobacterium faecale* abundance (149), and *Streptococcus*/*Escherichia-Shigella* levels correlate with disease severity (46). Macrovascular invasion (MVI) in HCC is marked by *Firmicutes* depletion and *Proteobacteria*/*Bacteroidetes* enrichment (150), particularly in HBV-related cases where *Prevotella_9* and *Megamonas* increase (150). Notably, *Bacteroides thetaiotaomicron* depletion distinguishes recurrence-prone HCC patients (151). Mendelian randomization identifies *Ruminococcaceae* and *Bacteroidetes* as protective against HCC development (152), underscoring the potential of microbiota-targeted interventions in precancerous stages to mitigate progression.

### 6.3 The diagnostic value of microbiota in HCC

RF analysis revealed that nine gut microbial genera, including *Elizabethkingia*, *Burkholderia_Caballeron-ia_Paraburkholderia*, *Klebsiella*, *Delftia*, *Faecalibaculum*, *Acetatifactor*, *Lactobacillus*, *Ruminococcaceae*_UCG-010, and *Stenotrophomonas*, could significantly distinguish the HCC group from the control group, with an AUC of 0.810. When these genera were combined with serum AFP levels, the AUC further improved to 0.980, demonstrating higher diagnostic efficacy (46). Additionally, 14 and 10 cross-dataset reproducible differential genera were identified in LC and HCC patients, respectively. RF models constructed based on these genera achieved AUCs of 0.820 and 0.900 for distinguishing cirrhosis and HCC in the training dataset and successfully achieved cross-regional validation (45). Studies based on ML algorithms further demonstrated that gut microbial biomarkers have high diagnostic accuracy in HCC subgroup classification, with an AUC of up to 0.940 (148). Peng et al. established a gut microbial diagnostic model, validating the potential of gut microbiota as a non-invasive tool for preoperative diagnosis of MVI (150). Through five-fold cross-validation, the study identified the optimal 30 microbial biomarkers and achieved an AUC of 80.64% in 75 early HCC samples and 105 non-HCC samples, demonstrating strong diagnostic capabilities for both early and advanced HCC (39). More importantly, the study successfully achieved cross-regional validation of microbial biomarkers in HCC patients from northwest and central China (39) (Table 2). The combination of gut microbial biomarkers with existing diagnostic methods, such as serum AFP, can significantly improve the diagnostic accuracy of HCC. In the future, validating these microbial biomarkers in larger, multi-regional clinical cohorts will help further enhance the stability and reliability of HCC diagnosis.

### 6.4 Mechanisms of the gut microbiota in HCC

**Figure 3:**
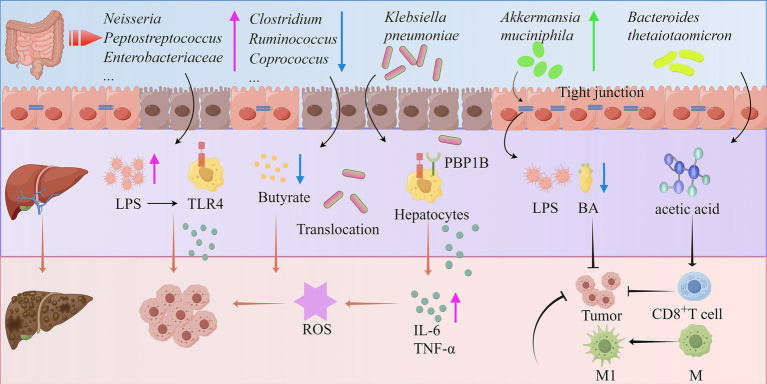
Mechanisms of the gut microbiota in the development of HCC (by Figdraw). ROS, Reactive oxygen species; IL-6, Interleukin-6; M1, Macrophage 1; M2, Macrophage 2; PBP1B, Penicillin binding protein 1B.

Recent studies have shown that gut microbiota dysbiosis plays a critical regulatory role in the pathological progression of HCC. The gut microbiota of patients with cirrhosis-related HCC exhibits characteristic changes, including a reduction in butyrate-producing bacteria (*Clostridium*, *Ruminococcus*, and *Coprococcus*) and an increase in LPS-producing bacteria (*Neisseria*, *Peptostreptococcus*, *Enterobacteriaceae*, and V*eillonella*). This imbalance in microbial metabolites may accelerate disease progression (153). LPS can activate TLR4 signaling in resident hepatocytes, stimulating the secretion of cytokines (IL-6 and TNF-α), leading to liver inflammation and oxidative damage (154, 155). *Klebsiella pneumoniae* promotes the development of precancerous lesions and HCC in mice by disrupting intestinal barrier integrity and translocating to the liver. Mechanistically, its PBP1B protein binds to TLR4 on HCC cells, thereby activating TLR4-mediated oncogenic signaling and driving tumorigenesis (156). Additionally, the gut microbiome transports microbial-associated molecular patterns and metabolites to the liver via the portal vein, thereby regulating HCC progression through the gut-liver axis (157) *Akkermansia muciniphila* enhances the efficacy of PD1 therapy by restoring gut barrier integrity to reduce LPS influx, suppressing the TLR2/NF-κB signaling pathway to diminish immunosuppressive m-MDSCs and M2 macrophages, while modulating cholesterol and BA metabolism (158). Notably, in viral hepatitis-related HCC patients, SCFA-producing bacteria are significantly enriched (148). Integrated analysis of the gut microbiome and tissue metabolome reveals that gut microbiota-derived acetate can be absorbed by the liver, providing energy support for tumor cell growth and proliferation, which may be an important mechanism for microbiota-mediated HCC recurrence (149). Particularly insightful is the finding that acetate derived from *Bacteroides thetaiotaomicron* can promote macrophage polarization toward a pro-inflammatory phenotype while enhancing T cell-mediated tumor cell killing, thereby inhibiting HCC progression (151). These groundbreaking findings collectively confirm that gut microbiota dysbiosis and the resulting barrier dysfunction and metabolite imbalance play a pivotal regulatory role in the pathological progression of liver cancer by promoting bacterial translocation and reshaping the immune microenvironment (Figure 3).

### 6.5 Treatment of the gut microbiota in HCC

#### 6.5.1 FMT

Following FMT, the abundance of beneficial gut bacteria such as *Lactobacillaceae*, *Bacilli*, and *Bacteroides* significantly increased, improving the diversity, richness, and evenness of the gut microbiota in cirrhotic rats, thereby alleviating liver fibrosis (143). Transplantation of fecal bacteria from wild-type mice or *Lactobacillus reuteri* into HCC mice elevated acetate levels and reduced IL-17A secretion, enhancing the anticancer effects in HCC mice (159). The absence of *Akkermansia muciniphila* has been associated with increased abundance of hepatic monocytic myeloid-derived suppressor cells. Notably, FMT-mediated reintroduction of *Akkermansia muciniphila* in Nlrp6^−/−^ mice restored intestinal barrier integrity and markedly attenuated hepatic inflammation and fibrosis (160).

#### 6.5.2 Probiotics

Probiotics, as important microbial agents for maintaining gut microbiota stability, include LAB strains such as *Lactobacillus* and *Bifidobacterium* (161). They can modulate the gut microbiota, stabilize the intestinal barrier, and mitigate carcinogenic toxicity, thereby influencing the development and progression of liver cancer (162). Studies have found that after 48 h of LAB treatment, the abundance of *Firmicutes*, *Bacteroidetes*, and *Actinobacteria* significantly increased in HCC mice, while the abundance of *Proteobacteria* was significantly lower than in untreated HCC groups (147). Furthermore, LAB, particularly *L. brevis* SR52-2 and *L. delbrueckii* Q80, exhibit antiviral properties that help improve gastrointestinal health in HCC patients (147).

#### 6.5.3 Diet

In a high-fructose diet-fed HCC mouse model, microbiota-derived acetate increased levels of glutamine and UDP-N-acetylglucosamine in HCC, enhancing protein O-GlcNAcylation and promoting HCC progression (163). Conversely, a 5:2 intermittent fasting regimen improved MASH and fibrosis and inhibited HCC development by activating hepatic PPARα and PCK1 (113) (Table 3).

These findings suggest that FMT, probiotics, and dietary interventions may exert beneficial effects on liver cirrhosis and HCC by modulating gut microbiota functionality. However, current research remains predominantly limited to animal studies, highlighting the need for further clinical validation to address existing gaps in translational applicability.

## 7 Expression characteristics, mechanistic research, and diagnostic-therapeutic applications of gut microbiota in drug-induced liver injury

### 7.1 Alteration of gut microbiota in drug-induced liver injury

Drug-induced liver injury (DILI), a leading cause of ALF and acute hepatitis globally (1), is a severe adverse drug reaction associated with medications such as anti-infectives, herbal products, and non-steroidal anti-inflammatory drugs (164, 165). Emerging evidence highlights the critical role of gut microbiota in DILI pathogenesis. Patients with DILI exhibit significant gut microbial dysbiosis, characterized by reduced richness and diversity (99), with distinct patterns across drug types. In acetaminophen (APAP)-induced models, APAP exposure increases Cyanobacteria and Deferribacteres while decreasing Firmicutes at the phylum level, and elevates *Bacteroides/Enterococcus* but depletes *Bifidobacterium/Lactobacillus* at the genus level (166, 167). Similar dysbiosis is observed in dietary supplement- or conventional drug-induced DILI, marked by reductions in *Acetobacteroides*, *Blautia*, and *Coprococcus* (99). Idiosyncratic DILI patients show *Bacillota* enrichment and *Bacteroidota/Verrucomicrobiota* depletion, with *Alloprevotella* dominance over *Eubacterium eligens* (168). Metronidazole (MNZ) exposure in mice reduces α-diversity and elevates *F/B* ratios alongside *Lactobacillus reuteri* abundance (169), though conflicting results on microbiota changes [e.g., Tulstrup et al. (170)] suggest dose- and duration-dependent effects. Mendelian randomization identifies *Oscillospira*, *Blautia*, and *Prevotella_7* as risk-associated taxa (171), while cisplatin and methotrexate models link *Proteobacteria*, *Enterococcus*, and *Collinsella* to liver injury (172, 173). These findings collectively underscore gut microbiota as a pivotal mediator in DILI progression.

### 7.2 Mechanisms of the gut microbiota in DILI

Thousands of drugs can induce direct, indirect, or idiosyncratic liver injury, with mechanisms that are complex and not yet fully elucidated. Studies have shown that the loss of intestinal barrier integrity leading to increased intestinal permeability may be one of the important mechanisms of DILI. APAP administration can upregulate the colonic epithelial chemokine (C-C motif) ligand 7, thereby mediating intestinal barrier dysfunction, which may be a key factor in APAP-induced hepatotoxicity (174). Additionally, metronidazole can disrupt the structure and function of the intestinal barrier, leading to gut microbiota dysbiosis and subsequent intestinal and liver injury (169). Numerous studies have demonstrated that the composition of gut microbiota is altered in DILI patients and animal models, manifesting as increased intestinal permeability, elevated LPS translocation, reduced SCFA production, and disrupted BA metabolic homeostasis (175). In a clinical trial, antithyroid drugs were found to increase fecal and serum LPS levels in patients, activating LPS-related signaling pathways and thereby inducing liver injury (176). In an APAP-induced mouse model of acute liver injury, ampicillin exacerbated APAP-induced liver injury by inducing gut microbiota imbalance and reducing butyrate levels (177). However, some studies have also suggested that gut microbiota metabolites may have protective effects on the liver. For instance, daidzein released by β-galactosidase from *Lactobacillus vaginalis* can inhibit Fdps-mediated hepatocyte ferroptosis, thereby ameliorating APAP-induced liver injury in mice (178). These findings indicate that the mechanisms by which gut microbiota regulates drug-related liver injury may be associated with intestinal barrier disruption, alterations in gut microbiota composition, and its metabolites. Future research should further explore the specific mechanisms of gut microbiota and its metabolites in liver injury and develop intervention strategies based on gut microbiota modulation, providing new targets for the prevention and treatment of drug-induced liver injury.

### 7.3 Treatment of the gut microbiota in DILI

The occurrence of DILI is accompanied by structural changes in the gut microbiota, and modulating the gut microbiota can effectively alleviate DILI. Compared to donor feces, oral fecal gavage enriches *Lachnospiraceae* and butyrate in the gut, mitigating APAP-induced ferroptosis through the AMPK-ULK1-p62 signaling pathway while simultaneously inducing mitochondrial autophagy and the Nrf2 antioxidant response, thereby effectively alleviating ALI in mice (179). Additionally, oral magnesium reduces APAP-induced liver injury by increasing the abundance of Bifidobacterium and inhibiting the production of the gut microbiota metabolite CYP2E1 (180). *Bacteroides vulgatus* exhibits probiotic effects *in vivo*, inhibiting the colonization of pathogenic microorganisms and alleviating APAP-induced oxidative stress and liver injury (181). Studies have also found that triptolide (TP) significantly disrupts gut microbiota composition, particularly reducing the relative abundance of *Lactobacillus rhamnosus* GG (LGG). Supplementation with LGG can reverse TP-induced hepatotoxicity by increasing bile salt hydrolase activity and reducing elevated conjugated BAs (182). Magnesium isoglycyrrhizinate treatment increases the abundance of the probiotic Lactobacillus, restores the intestinal barrier, and ameliorates methotrexate-induced liver injury (200). Furthermore, a randomized clinical trial demonstrated that probiotics may help alleviate drug-induced liver dysfunction in patients with depression (183). These studies indicate that FMT and probiotic treatments can restore gut microbiota homeostasis, enhance intestinal barrier function, and improve liver function parameters, thereby effectively mitigating the onset and progression of DILI. These findings provide new insights and approaches for the treatment of DILI. At the same time, attention should be paid to individual differences and the complexity of gut microbiota changes to achieve more precise and personalized treatments.

## 8 Other liver diseases and gut microbiota

In recent years, studies have revealed that, in addition to common liver diseases, autoimmune and genetic liver diseases such as primary biliary cholangitis (PBC), primary sclerosing cholangitis (PSC), autoimmune hepatitis (AIH), and Wilson’s disease (WD) are also closely associated with gut microbiota dysbiosis. Among these, PBC, as a progressive autoimmune liver disease, often presents with subtle early clinical manifestations. Research indicates that the albumin-bilirubin (ALBI) score and its grading system can effectively assess disease progression and prognostic risk in PBC patients. Notably, ALBI grade 1 patients exhibit higher gut microbiota α-diversity and ecological balance, with a significant predominance of *Clostridia* and *Lachnospira* genera. ML models have identified *Lachnospira* as a key biomarker for distinguishing different ALBI grades (184). In terms of therapeutic interventions, UDCA treatment can induce gut microbiota remodeling in PBC patients, with a particularly prominent expansion of *Bacteroides* in high-*Clostridia* microbiota. This microbial modulation may enhance the clinical response to UDCA by restoring gut homeostasis (185).

PSC is a chronic cholestatic liver disease characterized by typical pathological alterations including abnormal liver enzymes, dysregulated bile acid metabolism, and altered gut microbiota composition. Patients with PSC exhibit significantly reduced α-diversity and markedly decreased species richness in their gut microbiota (186). An observational study of 43 Czech PSC patients demonstrated significant upregulation of fecal bacterial genera including *Haemophilus*, *Rothia*, *Clostridium*, *Enterococcus*, *Streptococcus*, and *Veillonella* compared to HC (187). Inverse variance weighted analysis further revealed that the relative abundance of *Eubacterium hallii* was positively associated with PSC risk, whereas *Clostridiaceae1* and L*achnospiraceae* families showed significant negative correlations with PSC susceptibility, suggesting their potential protective roles (188). In clinical interventions, single FMT administration in 10 PSC patients resulted in ≥50% reduction in alkaline phosphatase levels in 3 cases. Notably, FMT not only significantly enhanced microbial diversity but also induced clinical remission of comorbid ulcerative colitis with efficacy comparable to biologic agents (189).

For AIH, the chronic liver inflammation process is closely linked to gut microecological imbalance. AIH patients exhibit a significant reduction in gut microbiota diversity, which not only alters microbial metabolic profiles but may also exacerbate liver inflammation by disrupting intestinal barrier integrity and immune regulatory functions (190). In the context of inherited metabolic liver diseases, WD patients experience copper metabolism disorders due to mutations in the ATP7B gene. 16S rRNA sequencing has shown that the abundance of *Selenomonaceae* and *Megamonas* genera in the gut of WD patients is significantly higher than in healthy individuals. This microbial abnormality may be pathologically associated with copper deposition and toxic damage in hepatocytes caused by impaired biliary copper excretion (191). Gut microbiota dysbiosis plays a crucial role in the development and progression of autoimmune and genetic liver diseases. Modulating the gut microbiota may provide new insights for the diagnosis and treatment of these diseases. Future research should further explore the specific mechanisms of gut microbiota in these liver diseases and develop gut microbiota-based early diagnostic and therapeutic approaches, thereby offering more precise and personalized treatment strategies for patients.

## 9 Summary and perspectives

This review explores the role of gut microbiota in various liver diseases, analyzing its applications in disease diagnosis and its contributions to disease progression. Studies have shown that patients with liver diseases exhibit a reduction in beneficial bacteria and an increase in potentially pathogenic bacteria within the gut, leading to dysbiosis of the gut microbiota. This imbalance further disrupts the integrity of the intestinal mucosal barrier, facilitating the translocation of bacteria and their toxins to the liver, creating a vicious cycle that exacerbates liver disease progression. Interventions such as FMT, dietary modifications, and oral administration of probiotics or prebiotics can effectively modulate the structure of gut microbiota, increase the abundance of beneficial bacteria, inhibit the growth of harmful bacteria, improve the gut microenvironment, alleviate hepatic inflammation, and protect hepatocytes from damage.

Despite the growing attention and extensive research on the relationship between gut microbiota and liver diseases, the gut microbiota is influenced by multiple factors, including dietary habits, geographic environment, host genetic background, age, technical variations, and pharmacological interventions. This has led to inconsistencies and limited comparability across studies regarding gut microbiota alterations. Current research primarily focuses on changes in microbiota composition, while the functional roles of microbiota, their metabolites, and their interaction mechanisms with the liver require further in-depth investigation. Future studies should aim to elucidate the specific molecular targets of gut microbiota in the pathogenesis of liver diseases and clarify the unique interactions between gut microbiota and different types of liver diseases. In recent years, research on microbial communities beyond the gut, such as oral microbiota, has also demonstrated their significant relevance to the onset, progression, and prognosis of liver diseases. This discovery provides a new perspective for liver disease research and may offer important insights for developing precision diagnostic tools and personalized treatment strategies based on microbial community characteristics. Additionally, research on gut microbiota modulation strategies should be expanded, optimizing the use of probiotics, prebiotics, and exploring novel microbiota transplantation methods. These efforts will provide more effective approaches for the comprehensive prevention and treatment of liver diseases, ultimately improving patient prognosis and quality of life.
